# A case report: A rare case of infant gastrointestinal canthariasis caused by larvae of *Lasioderma serricorne* (*Fabricius,* 1792) (Coleoptera: Anobiidae)

**DOI:** 10.1186/s40249-016-0129-6

**Published:** 2016-05-03

**Authors:** Xi Sun, Li-Fu Wang, Ying Feng, Hui Xie, Xiao-Ying Zheng, Ai He, Md Robiul Karim, Zhi-Yue Lv, Zhong-Dao Wu

**Affiliations:** Department of Parasitology, Zhongshan School of Medicine, Sun Yat-sen University, Guangzhou, 510080 China; Key Laboratory of Tropical Disease Control, Ministry of Education, Guangzhou, 510080 Guangdong China

**Keywords:** Canthariasis, *Lasioderma serricorne*, Larvae, *COX1*, 18S rRNA

## Abstract

**Background:**

Canthariasis is a disease of humans caused by the infestation of beetle larvae. It is the second important insectal disease after myiasis. Several species of beetles are reported to cause the disease in gastrointestinal tract, urogenital system, nasal sinuses, ears and faces of mammals. The cigarette beetle *Lasioderma serricorne* is a widespread and destructive pest that usually feeds on tobacco, tea, beans, cereal grains, and animal and plant specimen. While there was no previous evidence of human infestation by this worm, we report the first case of *L. serricorne* infestation in a baby girl in China.

**Case presentation:**

Here the case, an eight-month-old baby girl with irritable feeling, rubbing eyes, history of contact with mud and eating oranges twice during five days before attendance, and having “worms” in her stool was admitted to the First Affiliated Hospital of Sun Yat-sen University, Guangzhou, China. The clinical examination revealed that the pulse rate, blood pressure and temperature were regular, and the examination of the head, neck, and chest were unremarkable. The stool specimens containing “worms” were sent to the Department of Parasitology, Zhongshan School of Medicine, Sun Yat-sen University. The worms were recovered, studied morphologically using naked eyes and anatomical lens, PCR analyzed targeting cytochrome oxidase subunit 1 (*COX1*) and 18S rRNA genes, examined by sequence analyses of the PCR products and finally classified by phylogenetic analysis to identify their species. Based on the findings, the worms were diagnosed as the larvae of *L. serricorne*.

**Conclusion:**

This report implies that the baby had an infestation with the larvae of *L. serricorne* in the gastrointestine. During contact with mud or eating oranges by the girl, worm eggs were swallowed into the stomach and resisted gastric acid digestion which eventually hatched into larvae and caused canthariasis. The 8 months girl had underdeveloped immune system which might facilitate the disease. This report implicates that *L. serricorne* can infest human accidentally and cause canthariasis that may lead to severe damage to infant and older patient upon involvement of important organs of the body. The patients once diagnosed having canthariasis should be treated in time.

**Electronic supplementary material:**

The online version of this article (doi:10.1186/s40249-016-0129-6) contains supplementary material, which is available to authorized users.

## Multilingual abstracts

Please see Additional file 1 for translation of the abstract into the six official working languages of the United Nations.

## Background

Insectal diseases such as myiasis, scoleciasis and canthariasis are the infestations of the living or dead tissues of live vertebrates by developing larvae of the insects. Canthariasis or scarabiasis is an important insectal disease in humans caused by the beetles (coleopteran insects) or their larvae [1]. Difference to the myiasis, this is a rare infestation with its several forms. Canthariasis, the second important insectal disease, can infect gastrointestinal tract, urogenital system, nasal sinuses, ears and faces of mammals. Several species of beetles, including *Tenebrio molitor, A. piceus*, *T. mauritanicus*, *T. versicolor*, *C. punctatus* and species of skin beetles (Coleoptera: Dermestidae) and ground beetles (Coleoptera: Carabidae) have been reported to cause canthariasis [2–7]. In 1946, Palmer reported a case of intestinal canthariasis caused by *T. molitor* in a man in the USA [6]. In 1965, Scott reported that the black carpet beetle, *A. piceus* caused nasal cantheriasis and cadelle beetle, *T. mauritanicus* and large carpet beetle, *T. versicolor* caused enteric cantheriasis in humans in the USA. Wilson and Judd recovered a carpet beetle larva (Coleoptera: Dermestidae) from the digestive tract of an English woman in 1956 [5]. Bhargava and Victor reported two cases of Carabid beetle (*Crasydactylus punctatus Guerin*) invasion of human ears in Sultanate of Oman in 1999 [1]. Majumder and Datta recovered adult dung beetles from stool samples of 18 children from rural northeast India in 2012 [4]. In a recent study by Smadi and associates, the drugstore beetle, *S. paniceum* and a beetle of the genus *Trogoderma* were observed to cause facial cantheriasis in a 29-year-old woman in Jordan [8].

In this case report, we described a rare case of canthariasis caused by the infestation of *Lasioderma serricorne* larvae in an 8-month baby girl admitted to the department of Parasitology, Zhong shan School of Medicine, Sun Yat-sen University, Guangzhou, China.

## Case presentation

The case is a baby girl aged 8 months. The girl was brought to the hospital because her mother found several worms in her stool for 4 times in 3 days. Her mother was asked to answer some questions regarding age, residence, history of contact with animals or mud, and standard of living. Complete medical examinations, including the blood pressure, pulse rate and temperature, and examination of the head, neck, chest, were done in the First Affiliated Hospital of Sun Yat-sen Uuniversity, Guangzhou, China. The clinical examination revealed that the pulse rate was regular. The temperature and blood pressure were also normal. The head, neck, and chest were unremarkable. The patient felt irritable and was rubbing her eyes. She has history of contact with mud and ate oranges twice during five days before attendance. During the illness of patient, larvae were found seven times in the stool. Approximately, 21 larvae were recovered from the stool by her parents. The stool specimen containing worms was sent to the Department of Parasitology, Zhongshan School of Medicine, Sun Yat-sen University, Guangzhou, China for identification. At the time of our re-visiting the girl, two days after attendance, her parents confirmed us that no larva was appeared during this period without medication.

**Fig. 1:**
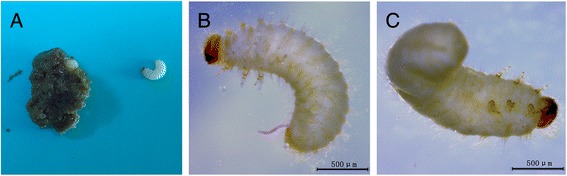
Examination the larvae of *L. serricorne*. Panel **a**: macroscopic examination of the larvae in stool. Panels **b**-**c**: microscopic examination of the larvae

The larvae were observed by the naked eyes for identifying the color and shape. The collected larvae were washed using phosphate buffered saline (PBS), and then examined by anatomical lens (Leica, Germany). Under the observation of naked eyes, the isolated larvae were subcylindrical, and bend in shape as “C” and creamy white in body color. Their heads were brown in color and their surfaces were convex and smooth. Upon examination under anatomical lens after cleaning by phosphate-buffered saline (PBS), the larvae were subcylindrical in shape, and creamy white in color. Their posture was as “C”. The larval body had wrinkles and different section size close and the head was tawny. The larvae were measured as 2–3 mm in length and ~ 0.5 mm in width, and their dorsal and ventral surfaces were full of plush. They had 3 pairs of feet on the belly near the head and the feet had 4 sections and nodes hocks curved claws (Fig. 1). All the characteristics of the larvae were very similar to the beetle larvae. To sum up, we hypothesized that the worm might be a member of beetles.

**Fig. 2:**
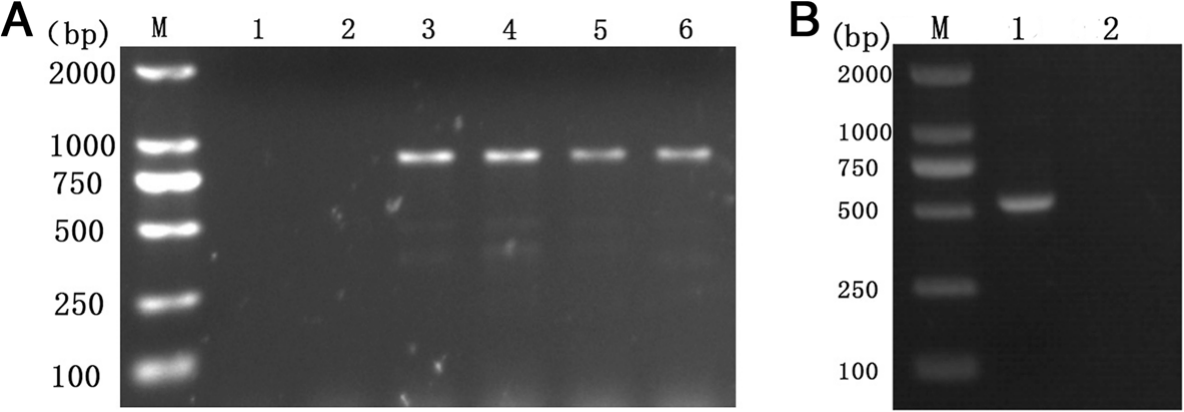
Representative electrophoresis results of *COX1* (**a**) and 18S rRNA (**b**) PCR products from larvae sample. Panel **a**- Lane M: DL2000 DNA marker; Lanes 1, 2: negative control (no DNA); Lanes 3, 4, 5, 6: *COX1* PCR product; and Panel B- Lane M: DL2000 DNA marker; Lane 1: 18S rRNA PCR product; Lane 2: negative control (no DNA)

Thereafter, the genomic DNA from three larvae was extracted using Hipure Tissue DNA Mini Kit (Magen, China) according to the manufacturer’s recommended protocol. Conventional PCR was conducted using the Green Master mix 2× (Promega, USA) and the primers, Pat (5′-TCCAATGCACTAATCTGCCATATTA-3′) and Jerry (5′-CAACATTTATTTTGATTTTTTGG-3′) for mitochondrial *COX1* gene of beetle larvae [9]. The following PCR cycling conditions were used for amplification: initial denaturation at 95 °C for 5 min; 30 cycles of 95 °C for 50 s, 55 °C for 50 s, 72 °C for 50 s; and a final extension at 70 °C for 10 min. Furthermore, 18S rRNA gene was PCR amplified using the Green Master mix 2× (Promega, USA) and the forward primer: TCGTCCACCTTGGTGACTCT and reverse primer: CTTCCGCGAACTCGGTGATA. The PCR consisted of 35 cycles at 94 °C for 30 s, 60 °C for 30 s, and 72 °C for 40 s, with an initial denaturation (94 °C for 5 min) and a final extension (72 °C for 10 min). The PCR products were analyzed by 1.0 % agarose gel electrophoresis, after staining with ethidium bromide. In the PCR analyses using larval DNA, species-specific products coinciding with the species-specific primers were successfully detected. Figure 2 (Panels a and b) shows the representative results of the PCR analyses. These results indicated that the larvae were beetle larvae.

In light of PCR analyses, further exploration was used to identify the larval species based on genetic characterization. Therefore, the amplified PCR products of the larval *COX1* and 18S rRNA genes were sequenced. Twenty PCRs were pooled and electrophoresed on a 1 % agarose gel. The expected band was excised, purified using Agarose Gel DNA Extraction Kit (TakaRa, Japan) and sequenced by company (Invitrogen). The obtained sequences were used for homology search using the BLAST program (http://blast.ncbi.nlm.nih.gov/Blast.cgi). The BLAST results revealed that the larval *COX1* and 18S rRNA gene sequences had high homology with published *L. serricorne COX1* gene (Accession No. DQ222030) and 18S rRNA gene (Accession No. AY748105), respectively. The alignments produced: a score = 1515 bits (820), expect = 0.0, identities = 824/826 (99 %), gaps = 0/826 (0 %) and strand = Plus/Plus for *COX1* and a score = 946 bits (512), expect = 0.0, identities = 521/525 (99 %), gaps = 2/525 (0 %) and strand = Plus/Plus for 18S rRNA. These findings suggested that the worms were *L. serricorne* larvae.

**Fig. 3:**
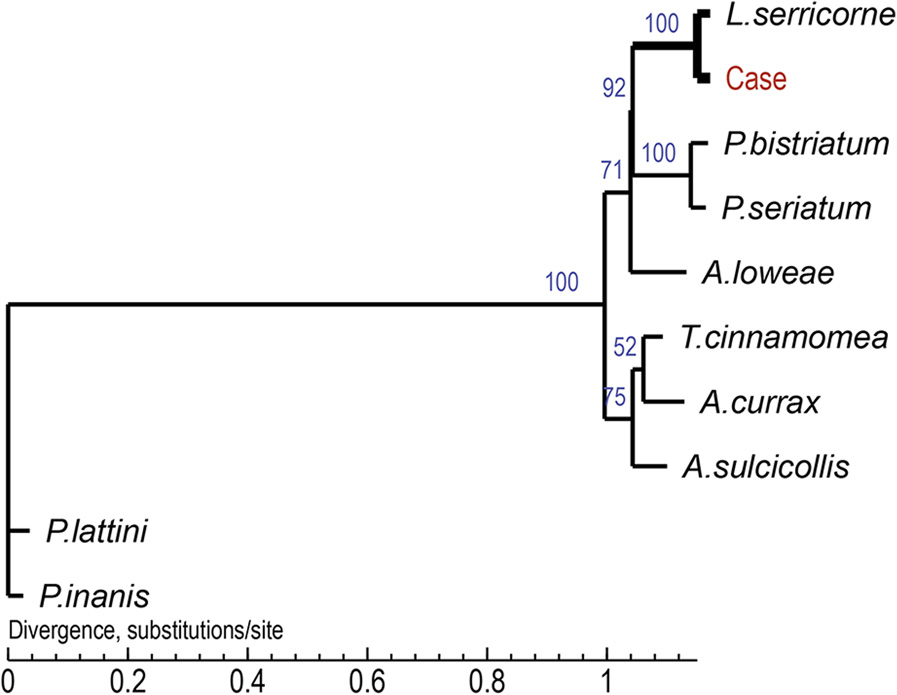
The Maximum Likelihood phylogenic tree of *COX1* in the sequence of case product and homologue species using PhyML (version 20120412). Multiple sequences alignment was performed by MUSCLE v3.8.31 with defaults

**Fig. 4:**
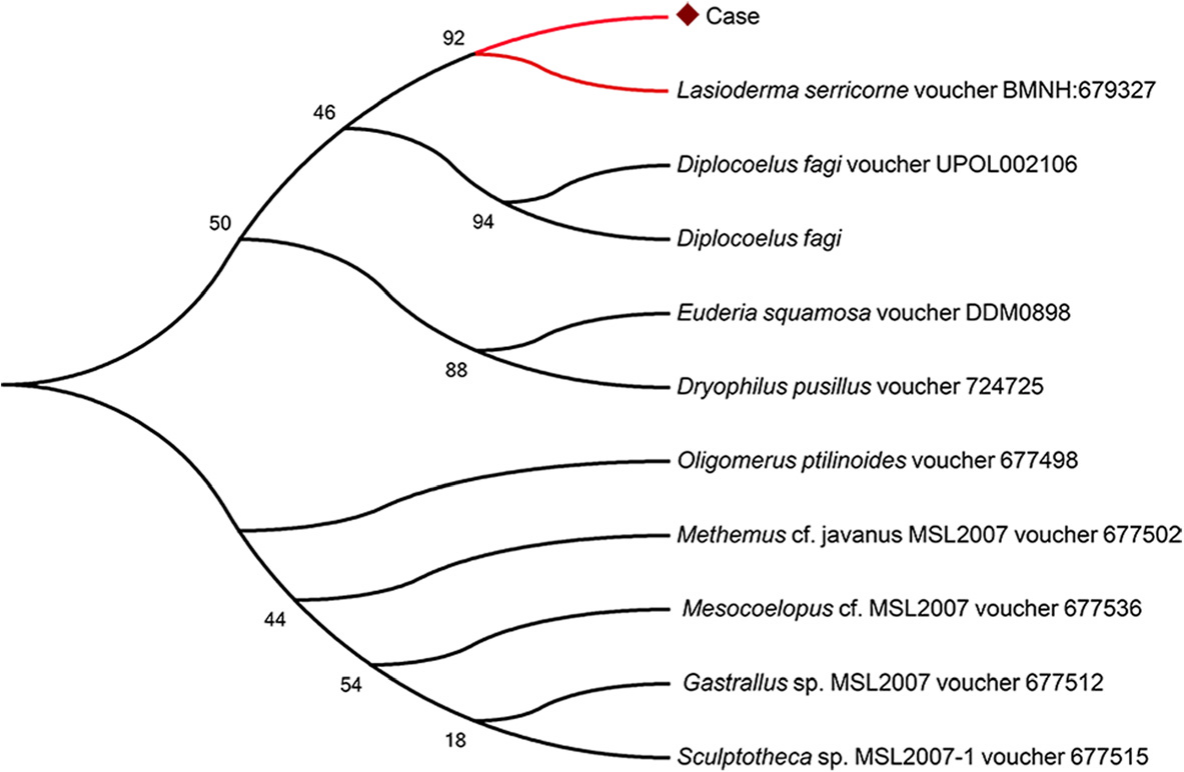
Phylogenetic analysis of 18S rRNA by Maximum Likelihood method. The evolutionary history was inferred by using the Maximum Likelihood method based on the Tamura-Nei model [12]. The tree with the highest log likelihood (−779.0954) is shown. The percentage of trees in which the associated taxa clustered together is shown next to the branches. Initial tree(s) for the heuristic search were obtained automatically by applying Neighbor-Join and BioNJ algorithms to a matrix of pairwise distances estimated using the Maximum Composite Likelihood (MCL) approach, and then selecting the topology with superior log likelihood value. The analysis involved 16 nucleotide sequences. All positions containing gaps and missing data were eliminated. There were a total of 467 positions in the final dataset. Evolutionary analyses were conducted in MEGA7. Multiple sequences alignment was performed by CLUSATLX2 with defaults

Finally, the obtained sequences were aligned with reference sequences downloaded from GenBank using the program ClustalX 1.83 (http://www.clustal.org/). The sequences from this study were compared with reference sequences using a Maximum likelihood method analysis of the aligned *L. serricorne* 18S rRNA sequences implemented in the program Mega 7 (http://www.megasoftware.net/). Boot-strap analysis was used to assess the robustness of clusters using 1,000 replicates. For the *COX1*, MUSCLE v3.8.31 with default parameters was used for multiple sequence alignment and the phylogenetic tree was constructed with the PhyML package using maximum-likelihood methods with default bootstrap and optimized calculation options. The phylogenetic analyses showed that both the observed *COX1* and 18S rRNA sequences clustered with *L. serricorne* reference sequences (Figs. 3 and 4). These results confirmed the larvae in this case as *L. serricorne*.

## Conclusions

Several forms of cantheriasis have been described in the previous reports. Thus far, rectal canthariasis caused by adults of the family Scarabaeidae in Sudan, enteric cantheriasis by cadelle beetle, *Tenebroides mauritanicus*, and large carpet beetle, *Trogoderma versicolor* and nasal cantheriasis by black carpet beetle, *Attagenus piceus* in USA, otic cantheriasis by adult *Crasydactylus punctatus* (Coleoptera; Carabidae) in Oman and facial cantheriasis by the drugstore beetle, *Stegobium paniceum*, and a beetle of the genus *Trogoderma* in Jordan have been reported [1–3, 8]. Besides, adult dung beetles were recovered from stool samples of 18 children from rural northeast India [4].

Herein, we confirmed a rare case of intestinal canthariasis caused by *L. serricorne* in an eight months old baby girl hospitalized in the First Affiliated Hospital of Sun Yat-sen Uuniversity, Guangzhou, China*.*

*L. serricorne*, also known as cigarette beetle, is a widespread and destructive pest that has a wide range of feeding habits, including tobacco, tea, beans, cereal grains, and animal and plant specimens [10, 11]. However, there is no report of human infection by this worm. To the best of our knowledge, this is the first case report of infant canthariasis caused by *L. serricorne* in China, let alone possibly in the world.

In this case, the patient was a baby girl aged eight months who presented white worms in her stool, and has the history of contact with mud and ate oranges twice during five days before attendance. We isolated the worms from stool specimens and identified by the naked eyes and under microscope. We observed that the larvae were subcylindrical and bend in shape, creamy white in body color and brown in head color, and the surface was convex and smooth. We presumed that these kinds of worms might be the larvae of some insects, as the baby’s poor personal hygiene and poor state of health indicating the risk of acquiring gastrointestinal myiasis and canthariasis. As we know, the gastrointestinal myiasis and canthariasis are caused by the swallowing of fly eggs or beetle eggs accidentally without chewing by the patients; it is thought that the baby might be infected by the insect eggs when she was contacting with mud or eating oranges and without being digested in the stomach.

On our examination by anatomical lens, the characteristics of the larvae were very similar to the beetle larvae: subcylindrical in shape, “C” like in posture and creamy white in color, 2–3 mm in length and ~ 0.5 mm in width, full of plush on the dorsal and ventral surface, 3 pairs of feet with having 4 sections and nodes hocks curved claws on the belly near to the head, wrinkles on the body and tawny head. To substantiate the morphological similarity of the larvae to beetles, we examined the *COX1* and 18S rRNA genes of the larvae by PCR amplification and the amplified products were identical to the expected size that illustrated the larvae as beetle larvae. Then we sequenced the *COX1* and 18S rRNA genes of the larvae and the sequences were used for homology search using the BLAST program which revealed the highest sequence similarity (99 %) of the larval *COX1* and 18S rRNA genes with published *L. serricorne COX1* gene (Accession No. DQ222030) and 18S rRNA gene (Accession No. AY748105), respectively. The phylogenetic analyses of the larval *COX1* and 18S rRNA gene sequences with reference sequences also revealed that the larvae belonged to *L. serricorne*. Thus, the clinical observation, morphological characteristics of the larvae, PCR findings, the genetic identity and phylogeny, all confirmed the isolated worms as *L. serricorne* larvae that caused canthariasis in the girl.

In summary, we reported that *L. serricorne* is a kind of beetle that can infest human accidentally and cause canthariasis. In this case, the patient was a girl aged 8 months who had underdeveloped immune system which might facilitate the disease. *L. serricorne* eggs, during contact with mud or eating oranges by the patient, were swallowed into the stomach and resisted gastric acid digestion. After entering into the intestine, the eggs hatched into larvae and caused canthariasis. The clinical symptoms of gastrointestinal canthariasis observed here were relatively light. However, canthariasis may cause severe damage to the patient (especially infant or the old people) if the larvae parasitize in some important organs of the body, such as skin and external auditory canal. The infants once diagnosed as canthariasis patients should be treated in time. It also suggests that the infants and immunocompromised persons should pay attention to personal hygiene and reduce the risk of infection with similar diseases. Furthermore, our study identified the beetle species of canthariasis in human by molecular biological methods, providing a final diagnosis method for the disease in the clinics.

### Ethics statement

This study was approved by the Human Research Ethics Committee of the Sun Yat-sen University in China and the committee’s reference number is [(2016) 025]. The parents of the patient received an explanation about the scope of the study, such as objectives, procedures, and potential risks, and signed an informed consent statement before inclusion in the study.

### Consent

Written informed consent was obtained from the patient for publication of this Case Study and any accompanying images. A copy of the written consent is available for review by the Editor-in-Chief of this journal.

## Additional file

Additional file 1: Multiligual abstracts in the six official working languages of the United Nations. (PDF 319 kb)
