# Supplementary material for: A case report: A rare case of infant gastrointestinal canthariasis caused by larvae of Lasioderma serricorne (Fabricius, 1792) (Coleoptera: Anobiidae)
Source: Infect Dis Poverty. 2016 May 3;5:34. doi: 10.1186/s40249-016-0129-6 (PMC4853848; doi:10.1186/s40249-016-0129-6)
Supplement: Additional file 1: — Multiligual abstracts in the six official working languages of the United Nations. (PDF 319 kb) [file 40249_2016_129_MOESM1_ESM.pdf]

Translation of the abstract into the six official working languages of the United Nations

تقرير حالة: حالة إصابة نادرة لطفل بالداء الذراحي المعوي الناجم عن يرقة خنفساء التبغ (*Lasioderma serricorne*)
(فبريسوس، 1792) (غمديات الأجنحة: الغفريات)

أكسي سون، لي - فو ونغ، يانج فينج، هوي أكسي، أكسايو – يانج زانج، آ هي، محمّد ربيع كريم، زي – يو أل في، تشونغ – داو وو

ملخّص

تقديم عام: الداء الذراحي مرض يُصيب الإنسان ناجم عن يرقة الخنفساء ويُعتبر ثاني أهمّ مرض تُسبّبه الحشرات بعد مرض النغف أو داء الدودة الحلزونية. وقد تمّ اكتشاف عديد الأنواع من الخنافس التي تُسبّب المرض على مستوى الجهاز الهضمي والجهاز البولي التناسلي والجيوب الأنفية والأذنين والوجه للإنسان. خنفساء التبغ هي حشرة مُدمّرة واسعة الانتشار وتتغذّى عادة على التبغ والشاي والفاصولياء والحبوب وعينات من النباتات والحيوانات. بما أنه لم تُسجّل أي حالة سابقة لإصابة بشرية ناتجة عن هذه الدودة، فنحن نُعلن عن الحالة الأولى الناجمة عن يرقة خنفساء التبغ لطفلة صغيرة في الصين.

تقديم الحالة: تُعاني طفلة صغيرة تبلغ من العمر ثمانية سنوات من الاضطراب كما تقوم بفرك عينيها، وبعد اللعب بالطين تناولت البرتقال مرتين في اليوم طيلة خمسة أيام قبل ذهابها إلى المستشفى وكما وُجدت "ديدان" في برازها، وتمّ أخذها إلى مستشفى فارست أفيلياتيد هوسبيتول لجامعة سان يات سين في قوانغتشو في الصين. وكشفت الفحوصات السريرية بأن معدل النبض وضغط الدم ودرجة الحرارة منتظمة وفحوصات الرأس والرقبة عاديّة. تمّ إرسال عينات البراز التي تحتوي على "الديدان" إلى قسم دراسة الطفيليات لمدرسة تشونغشان للطب، جامعة سان يات سين، أين تمّ جمع الديدان ودراسة شكلها باستعمال العين المجردة والعين التشريحية وطريقة تفاعل البوليميريز المتسلسلPCR التي تستهدف جينة COX1 وجينات 18S rRNA. وبعد انتهاء عمليّة PCR يتمّ تحليل أجزاء الحمض النووي المتحصل عليها وتصنيفها باستعمال التحليل الفيلوجيني لتحديد نوع هذه "الديدان". وبناءً على النتائج المتحصّل عليها، تبين أن هذه الديدان هي يرقات خنفساء التبغ *L. serricorne*.

خاتمة: من خلال هذا التقرير تبيّن أن الطفلة أُصيبت بالداء الذراحي المعوي الناجم عن يرقة خنفساء التبغ *L. serricorne*، وهذا خلال اللعب بالطين وتناولها للبرتقال، حيث تم ابتلاع بيض الديدان لتصل إلى المعدة أين يُحتمل أن يفقس البيض ليتحوّل إلى يرقات والتي كانت السبب في الإصابة بالداء الذراحي. الفتاة ذات الثماني سنوات صغيرة السن ولذا سيكون نظام المناعة لديها ضعيف جدّا ومن المحتمل أن يكون هذا ما سهّل المرض. تبيّن من خلال هذا التقرير بأن خنفساء التبغ من الممكن أن تُصيب الإنسان عن طريق حادثة لتتسبّب له في الإصابة بالداء الذراحي والذي قد يُؤدي إلى أضرار جسيمة للرضع والكبار من خلال إصابة أعضاء مهمّة من الجسم، لذا عند تشخيص الإصابة بالمرض ينبغي العلاج فورا.

Translated from English version into Arabic by Zeineb Trabelsi, through

TRANSLATORS WITHOUT BORDERS

一例罕见的因烟草甲虫幼虫(Fabricius, 1792)（鞘翅目：窃蠹科）引起的婴儿胃肠道甲虫病

孙希, 王立富, 冯英, 解辉, 郑小英, 何蔼, Md Robiul Karim, 吕志跃, 吴忠道

摘要

引言：甲虫病是仅次于蝇蛆病的重要昆虫病，因甲虫幼虫感染人而发病。已报道有几种甲虫可以通过感染哺乳动物胃肠道、泌尿生殖系统、鼻窦、耳、面部等而引发疾病。烟草甲虫是广泛存在并具有破坏力的昆虫，以烟草、茶叶、豆类、谷粒、动物以及其他各类植物为食。然而，目前并没有关于烟草甲虫幼虫感染人的明确报道，因此，这是发生在中国的烟草甲虫幼虫感染女婴病例的首次报道。

病例介绍：该病例为一个年龄为 8 个月的女婴，就诊前 5 天内有泥土接触史并曾食用过柑橘，具有烦躁不安、经常揉眼等症状，父母发现婴儿粪便中出现幼虫，故到中国广州中山大学附属第一医院进行检查。临床检查显示其脉搏、血压及体温均正常，头部、颈部、胸部检查亦无明显变化。包含幼虫的粪便被送往中山大学中山医学院寄生虫学教研室进行鉴定。幼虫被收集，并通过肉眼和解剖镜对其形态学特征进行观察。同时，PCR 分析幼虫的细胞色素氧化酶亚基 1（*COX1*）和 18S rRNA 基因，对 PCR 产物进行测序，再通过进化树分析归类其具体物种。基于以上实验手段，结果诊断该幼虫为烟草甲虫幼虫。

结论：该病例报道显示婴儿胃肠道中有烟草甲虫幼虫感染。婴儿接触泥土和使用柑橘期间，可能误将虫卵吞入腹中，虫卵在腹中抵抗胃酸的消化并最终孵出了幼虫从而引发甲虫病。8个月的婴儿因免疫系统不成熟也可能是该病发生的重要原因。该病例报道提示，烟草甲虫可以经偶然途径感染人并引起甲虫病，通过影响重要机体器官而给婴儿和老龄病人带来严重危害。病人一旦发现甲虫病应及时就诊。

Translated from English version into Chinese by Li-Fu Wang

Rapport de cas : Un cas rare de canthariasis (ou canthariase) de grippe intestinale infnntile dû à des larves de *Lasioderma serricorne* (Fabricius, 1792) **(**Coleoptera: Anobiidae)

Xi Sun, Li-Fu Wang, Ying Feng, Hui Xie, Xiao-Ying Zheng, Ai He, Md Robiul Karim, Zhi-Yue Lv, Zhong-Dao Wu

Résumé

Origine: Canthariasis ou canthariase est une maladie touchant les êtres humains et qui est causé par des larves de coléoptères. C'est la deuxième importante maladie causée par des larves d'insectes venant après celle de la "myiase ». Plusieurs espèces de coléoptères ont été signalées comme pouvant causer la maladie dans les appareils gastro-intestinal et urogénital, les sinus, les oreilles et les têtes des mammifères. La « cigarette beetle » ou coléoptère (scarabée) de la cigarette (*Lasioderma serricorne*) est un insecte nuisible et très répandu qui se nourrit de tabac, de thé, de haricots, de graines de céréales, ou de spécimens d'origine végétale ou animale. Bien que ce ver n'ait jamais été précédemment signalé sur des êtres humains, nous faisons rapport d'un premier cas d'infestation du *l. serricorne* sur une toute petite fille en Chine.

Présentation du cas . Voici le cas : une toute petite fille de huit mois, admise dans le centre hospitalier affilié à l'université de Sun Yzt-sen, Guangzhou en Chine, présente des signes d'irritabilité, à les yeux irrités et rouges, est rentrée en contact avec de la boue et a mangé des oranges à deux reprises pendant cinq jours avant son entrée à l'hôpital, et qui a des « vers » dans ses selles. L'examen clinique a révélé que le pouls, la tension et la température étaient réguliers et qu'aucune anomalie n'avait été décelée lors des examens de la tête, du cou et de la poitrine. Les échantillons de selles contenant les "vers" ont été envoyés au département de parasitologie, à l'école de médecine de Zhougshan, université de Sun Yat-sen. Les vers ont été récupérés et leurs morphologies ont été éxaminées à l'oeil nu et à la loupe. Afin d'identifier leurs espèces, la procédure de réaction de polymérisation en chaine (RPC) a analysé des gènes de cytochrome oxidase subunit 1 (COX1) and 18S rRNA, examinés à travers d'analyses séquentielles de produits PCR, et catégorisés par la suite par une analyse phytogénétique. Les résultats ont établi la présence de vers de la catégorie *de larves de L.serricorne*.

Conclusion : Ce rapport démontre que l'enfant a eu une infestation de larves du *L.serricorne* dans l'intestin. Pendant le contact avec de la boue ou l'ingestion d'oranges, les œufs des larves ont été avalés dans l'estomac, ont résistés au mécanisme de digestion et à la production d'acide gastrique et, ont donné naissance à des larves et provoqués la canthariase. La petite fille de huit mois avait un système immunitaire non développé ce qui a facilité l'apparition de la maladie. Ce rapport démontre que les êtres humains peuvent être infestés accidentellement par le *L.serricorne* et contractés la canthariase qui peut conduire à des dommages importants sur des enfants ou sur des patients plus agés, puisque cette maladie affecte des organes importants du corps. Tous les patients, atteints de canthariase doivent être soignés à temps.

Translated from English version into French by veromarie, through

Описание клинического случая: Редкий случай желудочно-кишечного кантариаза, вызванного личинками *жука*

табачного Lasioderma serricorne (Fabricius, 1792) (Жесткокрылые: Точильщики)

Кси Сунь, Ли-Фу Ванг, Йин Фенг, Хуи Ксие, Ксяо-Йинг Женг, Ай Хе, ДМ Робиул Карим, Жи-Йуе Лв, Жонг-Дао Ву

Краткий обзор

Исходные данные: Кантариаз - это болезнь, поражающая человека, вызванная инвазией личинок жуков. Это вторая по значимости болезнь, вызываемая насекомыми после миаза. Стало известно, что некоторые виды жуков могут вызывать болезнь в желудочно-кишечном тракте, урогенитальной системе, придаточных полостях носа, ушах и лицах млекопитающих. Жук табачный *Lasioderma serricorne* - широко распространенное насекомое-вредитель, паразитирующее на табаке, чае, бобовых, злаковом зерне, а также на других видах растений и некоторых видах животных. Несмотря на то, что ранее не было случаев инвазии этим червем человека, мы сообщаем о первом случае инвазии *L. serricorne* маленькой девочки в Китае.

Описание случая: Восьмимесячная девочка была госпитализирована в Первую клиническую больницу университета Сунь Ятсена, Гуанджоу, Китай. У нее наблюдалась болезненная чувствительность, наличие "червей" в стуле, и она постоянно терла глаза. В течение пяти дней до посещения врача она ела апельсины дважды в день и контактировала с грязью. Клиническое обследование показало, что частота пульса, кровяное давление и температура были в норме, а обследование головы, шеи и груди не показало ничего примечательного. Образцы стула, содержащие "червей" были отправлены в отдел паразитологии Медицинской Школы Жонгшань Университета Сунь Ятсена. Черви были извлечены, изучены морфологически невооруженным глазом и с помощью анатомической линзы, подвержены анализу ПЦР, нацеленному на цитохромоксидазу - субъединицу 1 (COX1) и на гены 18S rRNA, исследованы посредством секвенирования продуктов ПЦР и, наконец, классифицированы при помощи филогенетического анализа для определения их вида. Основываясь на полученных данных, черви были диагностированы как личинки *L. serricorne.*

Заключение: Данный отчет заключает, что желудочно-кишечный тракт ребенка был инвазирован личинками *L. serricorne*. Во время контакта с грязью или при поедании апельсинов девочка проглотила яйца червей, которые попали в желудок и устояли перед воздействием кислоты желудочного сока, затем из них вылупились личинки, которые и вызвали кантариаз. Иммунная система восьмимесячной девочки была еще слаборазвита, что могло способствовать болезни. Данный отчет предполагает, что *L. serricorne* может случайно инвазировать человека и вызвать кантариаз, который может привести с серьезному повреждению организма младенцев и пожилых, поскольку поражает важные органы тела. Пациентам, которым поставлен диагноз "кантариаз", необходимо своевременное лечение.

Translated from English version into Russian by Natallia Lupik, through

TRANSLATORS
WITHOUT BORDERS

Un informe de caso: Un raro caso de cantariasis gastrointestinal infantil producida por larvas de *Lasioderma serricorne* (Fabricius, 1792) (Coleoptera: Anobiidae)

Xi Sun, Li-Fu Wang, Ying Feng, Hui Xie, Xiao-Ying Zheng, Ai He, Md Robiul Karim, Zhi-Yue Lv, Zhong-Dao Wu

Resumen

Antecedentes: La cantariasis es una enfermedad de los seres humanos que se produce por una infección por larvas de escarabajo. Es la segunda enfermedad producida por insectos más importante luego de la miasis. Se ha reportado que varias especies de escarabajos producen la enfermedad en el tracto gastrointestinal, el sistema urogenital, los senos nasales, oídos y cara de mamíferos. El escarabajo

del tabaco *Lasioderma serricorne* es una peste generalizada y destructiva que normalmente se alimenta del tabaco, té, habichuelas, granos de cereal y plantas y animales. Si bien no hubo evidencia anterior de infección en humanos por este gusano, reportamos el primer caso de infección por *L. serricorne* en una bebé en China.

Presentación del caso: He aquí el caso: una bebé de ocho meses de edad con irritabilidad, que se frota los ojos, con antecedentes de contacto con lodo y de haber comido naranjas dos veces en los cinco días previos a su asistencia y que tiene “gusanos” en las heces, fue internada en el Hospital First Affiliated de la Universidad de Sun Yet-sen en Guangzhou, China. El examen clínico reveló que el pulso, la presión sanguínea y la temperatura eran normales, y el examen de cabeza, cuello y tórax fue sin mayores hallazgos. Las muestras de heces que contenían “gusanos” se enviaron al Departamento de Parasitología de la Escuela de Medicina Zhongshan de la Universidad Sun Yat-sen. Se recuperaron los gusanos, se estudió su morfología a ojo desnudo y con lente anatómica, se analizó la focalización de los genes de la subunidad 1 de la oxidasa del citocromo (CODX) y 18S ARNr por PCR, se examinaron los productos de la PCR por análisis de secuencia y finalmente se los clasificó por análisis filogenético para identificar la especie. Con fundamento en los hallazgos, los gusanos fueron diagnosticados como larvas de *L. serricorne*.

Conclusión: Este informe implica que la bebé había sido infectada por larvas de *L. serricorne* en el tracto gastrointestinal. Durante el contacto con el lodo o al comer naranjas, los huevos de los gusanos fueron tragados al estómago resistiendo la digestión de los ácidos gástricos y eventualmente se transformaron en larvas que produjeron la cantariasis. La bebé de 8 meses tenía un sistema inmune subdesarrollado lo que podría facilitar la enfermedad. Este informe implica que *L. serricorne* puede infectar accidentalmente a seres humanos y producir cantariasis que puede resultar en daño severo a bebés y pacientes mayores al involucrar los órganos importantes del cuerpo. Una vez diagnosticados con cantariasis los pacientes deberían ser tratados de manera oportuna.

Translated from English version into Spanish by Maria Alejandra Aguada, through

TRANSLATORS
WITHOUT BORDERS
